# Reference values of normal liver stiffness in healthy children by two methods: 2D shear wave and transient elastography

**DOI:** 10.1038/s41598-020-64320-w

**Published:** 2020-04-29

**Authors:** Cristina Oana Mărginean, Lorena Elena Meliţ, Dana Valentina Ghiga, Maria Oana Săsăran

**Affiliations:** 1 Department of Pediatrics, “George Emil Palade” University of Medicine, Pharmacy, Science, and Technology of Târgu Mureș, Gheorghe Marinescu Street no 38, Târgu Mureș, 540136 Romania; 2 Department of Medical Informatics and Biostatistics, “George Emil Palade” University of Medicine, Pharmacy, Science, and Technology of Târgu Mureș, Gheorghe Marinescu Street no 38, Târgu Mureș, 540136 Romania; 3 Department of Pediatric Cardiology, “George Emil Palade” University of Medicine, Pharmacy, Science, and Technology of Târgu Mureș, Gheorghe Marinescu Street no 38, Târgu Mureș, 540136 Romania

**Keywords:** Paediatric research, Medical research

## Abstract

TE and 2D-SWE are well-documented in studies performed on adults, but those on pediatric patients are limited. The aim of this study was to establish pediatric reference values for liver stiffness using two elastography methods: 2D-SWE and TE. We performed an observational study on 206 healthy children. All children underwent anamnesis, clinical exam, laboratory tests, US exam, TE and 2D-SWE for liver stiffness assessment. The mean liver stiffness value by 2D-SWE for all children was 3.72 ± 0.48 kPa. The mean values ranged between 3.603 ± 0.2678 kPa (3–5 years of age) and 3.774 ± 0.4038 kPa (9–11 years). The reference values varied between 4.1386 kPa (3–5 years of age) and 4.88 kPa (12–15 years). The mean liver stiffness value by TE was 3.797 ± 0.4859 kPa. The values ranged between 3.638 ± 0.4088 kPa (6–8 years of age) and 3.961 ± 0.5695 kPa (15–18 years). The cutoff values varied from 4.4064 kPa (3–5 years of age) to 5.1 kPa (15–18 years). We found a significant positive correlation between E Median values by TE and age [95% CI: 0.1160 to 0.3798, r = 0.2526, p = 0.0002]. Our findings revealed that the mean values of liver stiffness for all children on 2D-SWE and TE were almost identical, 3.72 ± 0.48 kPa versus 3.797 ± 0.4859 kPa.

## Introduction

Hepatic and hepatobiliary disorders are common causes of morbidity and mortality in children^1^. Among the most frequent causes of chronic hepatic disease in children, we recall viral chronic hepatitis (B, C and D), autoimmune hepatitis, non-alcoholic steatohepatitis (NASH), metabolic disorders, drug-induced hepatitis or intoxications etc^2–4^. Long-term complications involve hepatic cirrhosis, malignancies or even hepatic failure^5,6^. Recent studies reported an increasing incidence of hepatic disorders in children most likely as a result of alarming rates of pediatric obesity that can lead to hepatopathies, such as non- alcoholic fatty liver disease (NAFLD) or even NASH^7^. The gold-standard of diagnosis for hepatic fibrosis for both children and adults is hepatic biopsy, but its main disadvantage consists in its invasivity^8,9^. Thus, hepatic biopsy is hindered by parents’ refusal in most of the cases, especially since other non-invasive diagnostic tools are available, such as transient elastography (TE), or Radiation Force Impulse Elastography (ARFI) and 2D-Shear Wave Elastography (2D-SWE) expressed in meter/second (m/s) or KiloPascals (KPa)^9,10^. Moreover, modern medicine trends support the use of non-invasive ultrasound-based diagnostic tools in all domains as well as in assessing hepatic fibrosis, increasing the patient’s satisfaction and comfort^11^.

TE, also called Fibroscan, it was initially used in adults for the assessment of hepatic fibrosis^12^, but recent studies proved its usefulness in children^13^. TE is a non-invasive and reproducible method in both adults and children^9,10^. The device used for TE assessment generates an elastic wave by means of a vibrator or special probe positioned on the thoracic wall, projected on the right liver lobe. Thus, this probe transmits a low-amplitude signal to the hepatic tissue inducing an elastic shear wave that propagates through this tissue, but also allowing measurement of wave velocity, expressed in kPa that is directly proportional to liver stiffness, which varies from 2.5 to 74 kPa^14^.

Real time elastography can be achieved by ARFI method that consists in shearing assessment of the examined tissue creating a circular wave in this tissue similar to a stone thrown into the water. For this exploration, the studies on healthy children or those with different disorders (e.g. chronic hepatitis, metabolic disorders, steato-hepatitis, etc.) are scarce without establishing reference values for this type of assessment^8–10^.

2D-SWE is a new ultrasound technique that can visualize in real time the tissue taking consideration the viscosity and elasticity of the examined tissue. In comparison to TE, this method assesses hepatic elasticity according to a color map in order to determine both qualitative and quantitative stiffness concomitantly expressed in m/s and KPa. Moreover, this method is not influenced by the respiratory phases and it does not require children to hold their breath, which is very difficult in small ages^10^. The region of interest or the targeted tissue can be modified in size and location in order to avoid artefacts as those that result from the large pulsatile vessels^15^. Moreover, real-time SWE provided the opportunity of adjustment during acquisition for assessing a more homogeneous area of liver tissue, ensuring the avoidance of excessive hepatic motion^15^. These properties are very helpful in decreasing the variability in measurements of liver stiffness with real-time SWE^16^.

For a proper assessment of liver fibrosis in children by these non-invasive methods, cutoff values for normal liver tissue in different age groups and gender are mandatory. Nevertheless, only a few studies were reported in children that aimed to assess liver stiffness through TE or 2D-SWE in different chronic disorders, and even fewer in healthy children^10,14,15,17–20^.

***The aim**** of this study is to assess the liver stiffness in healthy children and to establish the cutoff values using two elastography methods: 2D-SWE and TE*.

## Results

### Liver stiffness cutoff value obtained by 2D-SWE

**Figure 1:**
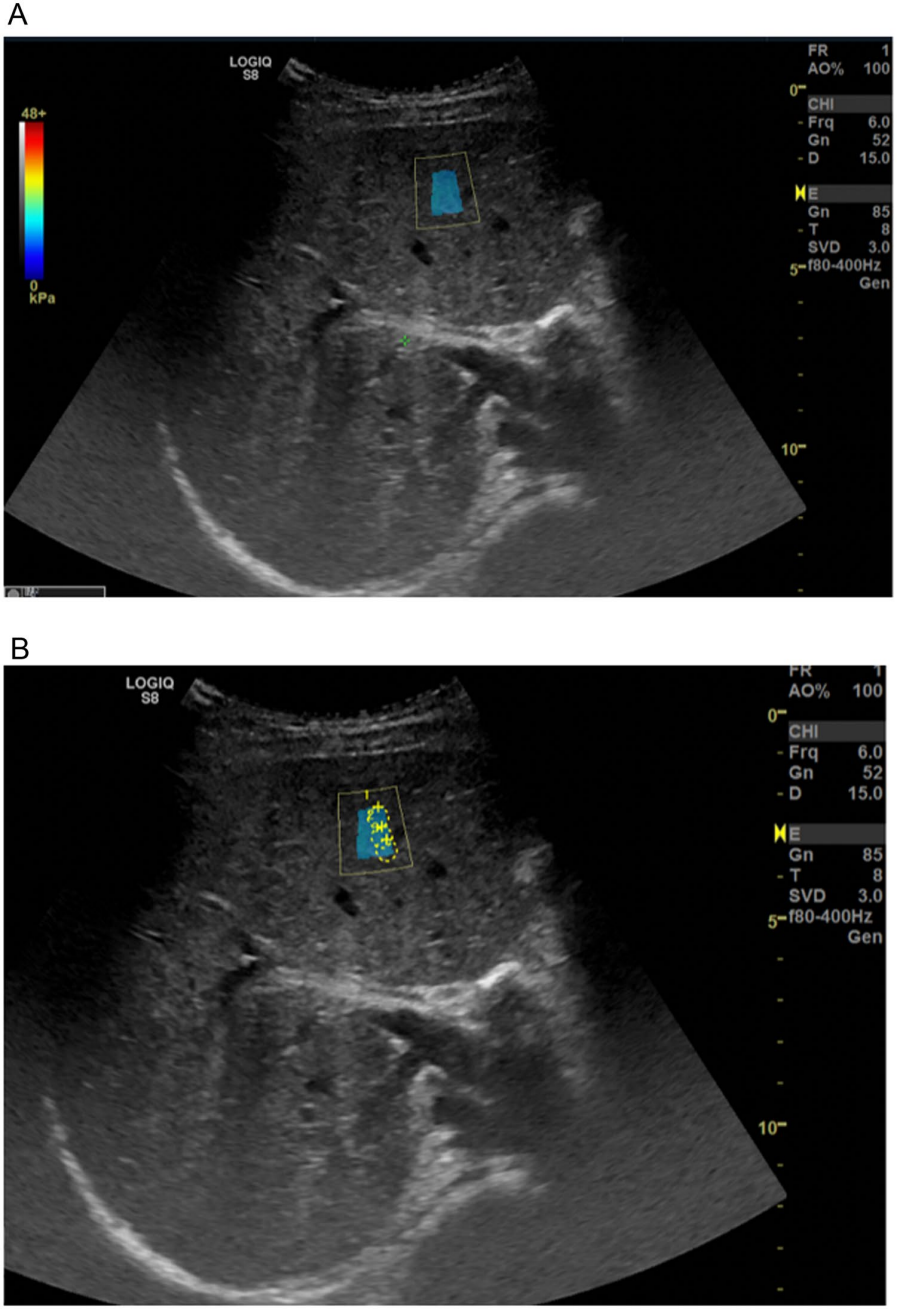
(**A**) Aspect of colored map on 2D-SWE (the picture was generate with Logiq Shear Wave Elastography S8 XDclear 2.0 software). (**B)** Regions of interest on 2D-SWE (the picture was generate with Logiq Shear Wave Elastography S8 XDclear 2.0 software).

In our study, the mean liver stiffness value measured by 2D-SWE (Fig. 1A,B) for all children was 3.72 ± 0.48 kPa. We divided our sample into 5 age groups taking into account different factors that might influence liver stiffness such as age, dietary habits, hormonal changes or metabolic factors. Thus, our sample was divided as it follows: 3 to 5 year of age – 14 children (6.69%), 6 to 8 years of age - 44 children (21.35%), 9 to 11 years of age 50 children (24.27%), 12 to 15 years of age - 63 children (30.58%), and between 15 and 18 years of age - 35 children (16.99%). Thus, our findings revealed mean values measured by 2D-SWE of 3.603 ± 0.2678 kPa in children with the age between 3 and 5 years; 3.749 ± 0.4202 kPa in the second age group (6–8 years of age); 3.774 ± 0.4038 kPa in children with the age between 9–11 years; 3.742 ± 0.5690 kPa in those between 12–15 years of age; and 3.624 ± 0.5172 kPa in the last age group (15–18 years). We found no significant differences between the age groups included in our study regarding the liver stiffness values measured by 2D-SWE (p = 0.5285) (Table 1). Similarly, the velocity (m/s) mean values measured by 2D-SWE for the same age groups were: 1.064 ± 0.0770 m/s in children between 3 and 5 years of age; 1.091 ± 0.0461 m/s in those between 6 and 8 years of age; 1.105 ± 0.0551 m/s in the third age group (9–11 years); 1.076 ± 0.1385 m/s in children with the age between 12–15 years of age; and 1.090 ± 0.0606 in the last age group (15–18 years of age); without significant differences between these age groups (p = 0.3104) (Table 1).

**Table 1:** Results of liver stiffness values (kPa) and velocity (m/s) by 2D-SWE on age groups.

Parameters	Variables	3–5 years (n = 14)	6–8 years (n = 44)	9–11 years (n = 50)	12–15 years (n = 63)	15–18 years (n = 35)	p value
E Median (kPa)	Mean ± SD	3.603 ± 0.2678	3.749 ± 0.4202	3.774 ± 0.4038	3.742 ± 0.5690	3.624 ± 0.5172	0.5285
	95% CI of mean	3.448–3.757	3.621–3.876	3.659–3.889	3.599–3.885	3.447–3.802	
	Cutoff value (Mean + 2 SD)	4.1386	4.5894	4.5816	4.88	4.6584	
V median (m/s)	Mean ± SD	1.064 ± 0.0770	1.091 ± 0.0461	1.105 ± 0.0551	1.076 ± 0.1385	1.090 ± 0.0606	*0.3104
	95% CI of mean	1.020–1.109	1.077–1.105	1.090–1.121	1.041–1.111	1.069–1.111	
	Cutoff value (Mean + 2 SD)	1.218	1.1832	1.2152	1.353	1.2112	

CI – confidence interval, 2D-SWE – 2D-Shear Wave Elastography, E – elasticity, kPa – Kilo Pascal, m/s – meter/second, n – number, SD - standard deviation, V – velocity, *test Kruskal-Wallis.

The cutoff values for E median obtained by 2D-SWE for these age groups were: 4.1386 kPa in the first group (3–5 years of age); 4.5894 kPa in the second group (6–8 years); 4.5816 KPa in children between 9 and 11 years of age; 4.88 kPa in those between 12 and 15 years of age; and 4.6584 kPa in children with the age between 15 and 18 years (Table 1).

Similarly, the cutoff values for V median assessed by 2D-SWE on the same age groups were: 1.218 m/s in subjects with the age between 3 and 5 years; 1.1832 m/s in the second age group (6–8 years); 1.2152 m/s in the third age group (9–11 years); 1.353 m/s in those with the age from 12 to 15 years; and 1.2112 m/s in the last age group (15–18 years) (Table 1).

The Anova test performed on age groups pointed no significant difference between the mean values for E median (kPa) for the assessed age groups, or the mean values of V median (m/s) for the same age groups, p > 0.05.

Regarding the differences of these values for genders, we noticed that the E median value measured by 2D-SWE was a little higher in boys (3.787 ± 0.4656 kPa) as compared to those in girls (3.669 ± 0.4794 kPa), but without statistical difference between genders, p = 0.0774. In our study we examined 114 girls (55.33%) and 92 boys (44.66%). The cutoff value was 4.7182 kPa in boys and 4.6278 kPa in girls (Table 2).

**Table 2:** Results of liver elasticity values (kPa) and velocity (m/s) by 2D-SWE according to gender.

Parameters	Variables	Girls (n = 114)	Boys (n = 92)	p value
E Median (kPa)	Mean ± SD	3.669 ± 0.4794	3.787 ± 0.4656	0.0774
	95% CI of mean	3.580–3.758	3.690–3.883	
	Cutoff value (Mean + 2 SD)	4.6278	4.7182	
V median (m/s)	Mean±SD	1.085 ± 0.06040	1.092 ± 0.1171	*0.0182
	95% CI of mean	1.074–1.096	1.067–1.116	
	Cutoff value (Mean + 2 SD)	1.2058	1.3262	

CI – confidence interval, 2D-SWE – 2D-Shear Wave Elastography, E – elasticity, kPa – Kilo Pascal, m/s – meter/second, n – number, SD - standard deviation, V – velocity, *test Kruskal-Wallis.

Contrariwise, the V median value measured by 2D-SWE was significantly higher in boys (1.092 ± 0.1171 m/s) than in girls (1.085 ± 0.06040 m/s), p = 0.0182 (Table 2). The cutoff value for V median was 1.3262 m/s in boys and 1.2058 m/s in girls (Table 2).

We obtained no significant differences between the mean values of E Median (kPa) in females versus males in none of the assessed groups (p = 0.8303/p = 0.3376/p = 0.0890/p = 0.4400/ p = 0.1499). Similarly, no significant differences were noticed for V median in the 5 age groups included in our study between the 2 genders (p = 0.3006/p = 0.7289/p = 0.9671/p = 0.1460/ p = 0.0992).

Regarding the age, we found higher values for E median as the age was smaller, therefore a reverse correlation between age and E median, but without statistical significance [95% CI: −0.1673 to 0.1139, r = −0.02725, p = 0.6974]. A similar reverse correlation was also noticed for V median, higher values being observed in younger children, also without significant differences [95% CI: −0.1842 to 0.09665, r = −0.04468, p = 0.5237].

### Liver stiffness cutoff obtained by TE

**Figure 2:**
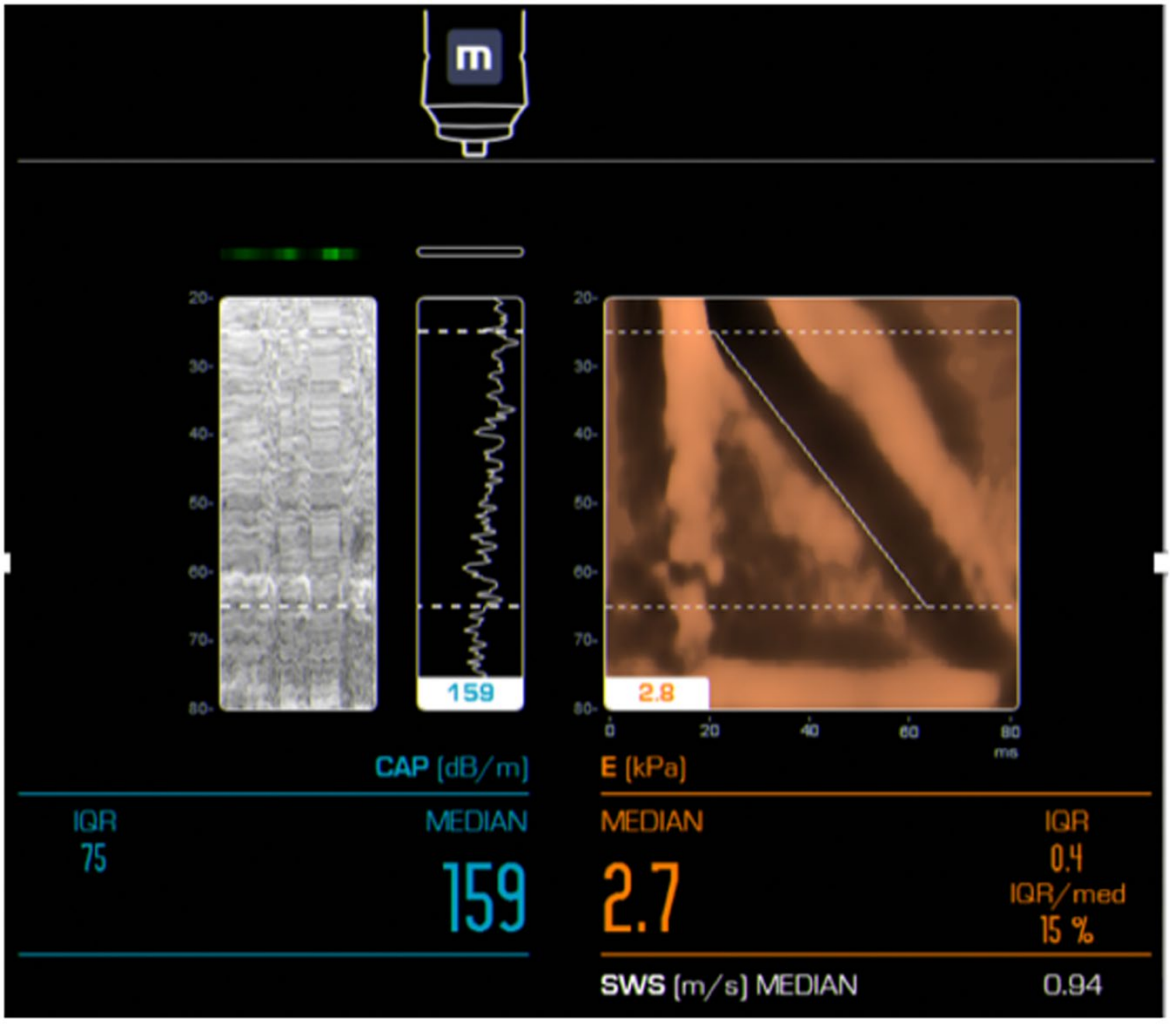
E median assessment on TE (the picture was drawn with Logiq *S8 XDclear 2.0 software and* FibroScan module IPX0).

In our study, the mean stiffness measured by TE (Fig. 2) for all the children was 3.797 ± 0.4859 kPa.

We assessed the mean values of E Median measured by the TE following the same age groups as for 2d-SWE. Thus, the mean values were: 3.657 ± 0.3747 kPa in children with the age between 3 and 5 years; 3.638 ± 0.4088 kPa in those between 6 and 8 years; 3.696 ± 0.4751 kPa in the third age group (9–11 years); 3.929 ± 0.4644 kPa in the fourth age group (12–15 years); and 3.961 ± 0.5695 kPa in the last age group (15–18 years of age). Thus, the cutoff values for E median measured by TE on age groups were: 4.4064 kPa for children between 3–5 years of age, 4.4556 kPa in the age group 6–8 years, 4.6462 kPa in children with the age between 9 and 11 years, 4.8578 kPa in those between 12 and 15 years, and 5.1 kPa in the last age group (15–18 years) (Table 3).

**Table 3:** Results of liver elasticity values (kPa) by TE on age groups.

Parameters	Age (n = 206)				
E Median Fibroscan (kPa)	3–5 years (n = 14)	6–8 years (n = 44)	9–11 years (n = 50)	12–15 years (n = 63)	15–18 years (n = 35)
Mean ± SD	3.657 ± 0.3747	3.638 ± 0.4088	3.696 ± 0.4751	3.929 ± 0.4644	3.961 ± 0.5695
Median ± standard error	3.745 ± 0.1002	3.735 ± 0.0616	3.700 ± 0.0672	3.900 ± 0.0585	3.950 ± 0.0963
95% CI of mean	3.441–3.874	3.514–3.762	3.561–3.831	3.812–4.046	3.765–4.157
25% Percentile	3.425	3.400	3.400	3.600	3.500
75% Percentile	3.925	4.000	4.025	4.300	4.200
Cutoff value (Mean + 2 SD)	4.4064	4.4556	4.6462	4.8578	5.1

CI – confidence interval, E – elasticity, kPa – Kilo Pascal, n – number, SD - standard deviation.

Applying the Dunn’s in order to compare the E Median values measured by TE between the assessed age groups, we found no significant difference between these groups for E median, except for a tendency towards statistical significance between 6–8 years group and 12–15 years group, p = 0.0057 (Table 4).

**Table 4:** The comparison of E Median between the assessed age groups (Dunn’s test).

Dunn’s Multiple Comparison Test (years)	Significant? p < 0.05?
3–5 vs 6–8	No
3–5 vs 9–11	No
3–5 vs 12–15	No
3–5 vs 15–18	No
6–8 vs 9–11	No
6–8 vs 12–15	Yes (p = *0.0057)
6–8 vs 15–18	No
9–11 vs 12–15	No
9–11 vs 15–18	No
12–15 vs 15–18	No

E – elasticity, vs – versus.

We found almost an identical value for liver stiffness measured by TE in boys (3.798 ± 0.5428 kPa) and girls (3.797 ± 0.4371 kPa), (p = 0.9324). The cut-off values obtained in our study were 4.8836 kPa in boys and 4.6712 kPa in girls (Table 5).

**Table 5:** Results of liver elasticity values (kPa) by TE according to gender.

Parameters	Gender	
E Median (kPa) TE (Fibroscan)	Female (n = 114)	Male (n = 92)
Mean ± SD	3.797 ± 0.4371	3.798 ± 0.5428
Median ± standard error	3.870 ± 0.04094	3.840 ± 0.05659
95% CI of mean	3.716–3.878	3.686–3.910
25% Percentile	3.500	3.400
75% Percentile	4.003	4.100
Cutoff value (Mean + 2 SD)	4.6712	4.8836

CI – confidence interval, E – elasticity, kPa – Kilo Pascal, m/s – meter/second, n – number, SD - standard deviation, TE – transient elastography.

Similar to 2D-SWE method, we obtained no significant differences between the mean values of E Median (kPa) by TE in females versus males in none of the assessed age groups (p = 0.1300/p = 0.0890/p = 0.0806/p = 0.5438/ p = 0.1241).

In terms of age, we encountered significantly higher values in older children, noticing a significant positive correlation between E Median values by TE and age [95% CI: 0.1160 to 0.3798, r = 0.2526, p = 0.0002].

### The liver stiffness values comparison – TE (Fibroscan) versus 2D-SWE

Comparing the E median value obtained by TE versus 2D-SWE we found significant differences only for older children, those included in the age groups 12–15 and 15–18 years, noticing higher values of liver stiffness measured by TE (Fibroscan) versus 2D-SWE method (p = 0.0455/p = 0.0065) (Table 6).

**Table 6:** Comparison of liver stiffness values by TE versus 2D-SWE.

Age (years)	E Median (kPa) TE (Fibroscan) Mean ± SD	E Median (kPa) 2D-SWE Mean ± SD	p value
3–5	3.657 ± 0.3747	3.603 ± 0.2678	0.6629
6–8	3.638 ± 0.4088	3.749 ± 0.4202	*0.4990
9–11	3.696 ± 0.4751	3.774 ± 0.4038	0.3798
12–15	3.929 ± 0.4644	3.742 ± 0.5690	0.0455
15–18	3.961 ± 0.5695	3.624 ± 0.5172	*0.0065

2D-SWE – 2D-Shear Wave Elastography, E – elasticity, kPa – Kilo Pascal, m/s – meter/second, n – number, SD - standard deviation, TE – transient elastography *test Mann-Whitney.

In females, the values of E Median obtained by TE were significantly higher as compared to those obtained by 2D-SWE (3.797 ± 0.4371 kPa versus 3.669 ± 0.4794 kPa), p = 0.0167. Contrariwise, in males we noticed no significant differences regarding the values of the same parameter obtained by the two methods, TE and 2D-SWE (3.798 ± 0.5428 kPa versus 3.787 ± 0.4656 kPa) (p = 0.9779).

Regarding the distribution of E Median values measured by the same two methods on group ages, we found significant differences in females aged between 12 and 15 years, in whom the values obtained by TE (Fibroscan) were significantly higher as compared to those recorded by 2D-SWE (3.964 ± 0.3902 kPa versus 3.702 ± 0.5746 kPa), (p = 0.0334), but also in older females, aged between 15 and 18 years, in whom we also noticed higher values for TE method than for 2D-SWE one (3.836 ± 0.3963 kPa, versus 3.524 ± 0.4070 kPa), p = 0.0073 (Table 7).

**Table 7:** The comparison between liver stiffness values (kPa) obtained by TE and 2D-SWE depending on age and gender.

Age	Gender	E Median (kPa) TE (Fibroscan) Mean ± SD	E Median (kPa) 2D-SWE Mean ± SD	p value
3–5 years	F	3.480 ± 0.3378	3.622 ± 0.3004	0.4605
	M	3.790 ± 0.3636	3.589 ± 0.2612	0.2243
6–8 years	F	3.735 ± 0.4241	3.816 ± 0.4654	*0.7660
	M	3.558 ± 0.3860	3.693 ± 0.3794	*0.3427
9–11 years	F	3.553 ± 0.4785	3.655 ± 0.3758	0.4559
	M	3.792 ± 0.4556	3.853 ± 0.4083	0.5850
12–15 years	F	3.964 ± 0.3902	3.702 ± 0.5746	*0.0334
	M	3.859 ± 0.5906	3.821 ± 0.5629	0.8317
15–18 years	F	3.836 ± 0.3963	3.524 ± 0.4070	0.0073
	M	4.323 ± 0.8302	3.913 ± 0.7019	0.2745

2D-SWE – 2D-Shear Wave Elastography, E – elasticity, F – female, kPa – Kilo Pascal, M – male, SD: standard deviation, *test Mann-Whitney.

Assessing the intra-observability of our study, we noticed that our data present excellent reliability. Thus, for E median, we obtained an ICC of 0.952 (95% CI:0.935–0.965, p < 0.0001), for V median an ICC of 0.978 (95% CI:0.957–0.987, p < 0.0001), while for E median by TE we found an ICC of 0.978, (95% CI:0.954–0.987, p < 0.0001). The intra-observability was assessed based on the measurements performed by a single examiner using different regions of interest on the colored maps stored initially in our device for each patient.

## Discussions

Liver fibrosis is a severe and common condition in both children and adults, and its correct evaluation is essential for treatment, prognosis and long-term follow-up. Liver biopsy remains the ‘gold-standard’ for the most accurate evaluation of liver fibrosis providing the possibility to identify also the grading of the liver condition along with specific markers for certain disorders or to reveal fatty infiltration within the hepatic tissue^21^. Elastography, a novel non-invasive US-based method consists in application of local mechanical compression on soft tissue along with the acquisition of strain images that trigger tissue responses^22,23^. Both TE and 2D-SWE are well-documented in studies performed on adults, but unfortunately the studies on pediatric patients are limited. Nevertheless, three main advantages of 2D-SWE were described with respect to TE: *i)* the use of B-mode image for guidance that allows the improvement of both variability in stiffness measurements and assessment of morphological modifications or identification of focal liver lesions; *ii)* the use of shear waves with greater bandwidths improves separation of fibrosis stages; *iii)* a real-time quantitative map of liver tissue stiffness^15,24,25^. On the other hand, TE proved its clinical applicability in patients with chronic hepatitis C, hemochromatosis, primary biliary cirrhosis, NASH, or human immunodeficiency virus with chronic hepatitis C co-infection^21^. Despite the fact that TE fails in differentiating between the initial stages of liver fibrosis, its sensitivity is high enough to delineate mild fibrosis from advanced staged and cirrhosis, especially regarding the treatment decision^21^. Our study expresses the important particularity of using both TE and 2D-SWE in assessing liver stiffness in healthy children.

To our knowledge, only a few studies focused on identifying reference values of normal liver stiffness in children using TE^26–28^ and ARFI^29–31^, but the scarcity is even more expressed regarding 2D-SWE method in control groups of healthy children^17,32,33^. Thus, the most recent study performed by Galina *et al*.^33^ on 202 healthy children using this method for the assessment of liver fibrosis showed that the values ranged between 2.7 and 5.76 kPa, with a mean value of 4.29 ± 0.59 kPa. Our study revealed a lower mean value in terms of 2D-SWE for normal liver stiffness, 3.72 ± 0.48 kPa, as compared to the previous study. This difference might be a result of the fact that the authors also included infants in their sample, in whom they found higher liver elasticity values similar to adolescents^33^, while our study assessed only children above the age of 3 years. Another difference is that they performed fewer measurements, at least 5 per patient, without obtaining the same number for every patient, in comparison to our study, which was based on 12 measurements for every patient. Regarding adolescents, our findings are consistent to those of Galina *et al*.^33^, revealing the highest values of liver stiffness in children above the age of 12 years: 4.88 kPa in children between 12 and 15 years of age, and 4.6584 kPa in those aged between 15 and 18 years. Similar to our study, the study of Galina *et al*.^33^ also used a General Electric US system. Other two studies that assessed normal liver stiffness in children on 2D-SWE used a different US system (Aixplorer, Supersonic Imagine, Aix-en-Provence, France) on a smaller number of pediatric subjects, and reported higher reference values for liver stiffness, 6.58 ± 1.46 kPa and 5.5 ± 1.3 kPa, respectively^17,32^. Both the system and the smaller sample size might have contributed to these differences between the previously mentioned studies and our findings.

On the other hand, Tokuhara *et al*. assessed liver stiffness in children using TE, proving age-dependent values in their study^26^. Thus, they noticed that median values increased with age: 3.4 kPa in preschool aged children, 3.8 kPa in elementary school children, and 4.1 kPa in adolescents. In our study, TE also revealed increasing values with age: 3.657 ± 0.3747 kPa in children with the age between 3 and 5 years; 3.638 ± 0.4088 kPa in those between 6 and 8 years; 3.696 ± 0.4751 kPa between 9–11 years; 3.929 ± 0.4644 kPa between 12–15 years; and 3.961 ± 0.5695 kPa in the last age group (15–18 years of age). Despite the fact that we identified no significant differences between these age groups, when correlating with age, we encountered significantly higher values in older children, noticing a significant positive correlation between E Median values on TE and age. The highest values for liver stiffness in adolescents might be explained by the important changes that occur in their body during this period, especially hormonal ones resulting in similar values to that of adults as reported by a study performed on healthy adults, 4.4 kPa^34^, which is similar to the values obtained in our group of adolescents.

Regarding gender, the studies reported in the literature are contradictory. Thus, certain studies based on ARFI found no influence of gender on liver stiffness^35,36^; while others that used TE underlined lower values in women, most-likely due to estrogen anti-fibrogenetic effect^37,38^. Moreover, 2D-SWE method also pointed out higher mean values of healthy liver elasticity in boys as compared to girls^33^. Similarly, our study also revealed higher values of liver stiffness in healthy males as compared to healthy females by both TE and 2D-SWE.

Based on the aforementioned facts, these two methods are very important in detecting liver fibrosis in patients with different liver disorders, being very useful since they are non-invasive and reproducibly. Nevertheless, cutoff values in healthy children must be clearly established on large cohorts since pediatric patients are completely different with respect to adults.

Our study has certain ***limitations***, among which the relatively small number of cases, especially below the age of 5 year; the lack of correlation with other biomarkers, such as Fibrotest or hepatic biopsy, even though it is not justified in healthy children; but also the fact that we used only one type of transducer. Nevertheless, our study has multiple ***strengths*** such as the assessment of liver stiffness by two novel elastography methods in healthy pediatric patients, the fact that all assessments were performed by a single experienced and skilled physician in pediatric US and elastography, the high number of measurement (12 in each patient), the accuracy of the statistical analysis, but also the cut-off values for liver stiffness that are essential as reference values when assessing children with different hepatic disorders.

To the best of our knowledge, this is the first study in Romania performed on such a big sample that assessed liver stiffness in healthy children by two novel non-invasive methods, TE and 2D-SWE. Moreover, our findings are also very important for the literature since the data regarding the assessment of liver stiffness in healthy children are scarce.

## Conclusions

2D-SWE and TE are two novel non-invasive elastography methods useful in assessing liver stiffness/fibrosis. Our findings revealed the mean liver stiffness value measured by 2D-SWE for all the children of 3.72 ± 0.48 kPa, with similar values for both stiffness and velocity independently of the age and gender according to 2D-SWE method. Thus, the cutoff values for stiffness ranged between 4.1386 for the youngest age group and 4.88 for children between 12 and 15 years of age; whereas for velocity the smallest cutoff value was encountered in children with the age between 6 and 8 years, 1.1832 m/s versus 1.353 m/s in those between 12 and 15 years of age. The mean values of liver stiffness for all children measured by TE was almost identical to that obtained by 2D-SWE, 3.797 ± 0.4859 kPa. The cutoff values of liver stiffness obtained by this method ranged between 4.4064 and 5.1 kPa, significantly increasing with age, but without differences between genders. Comparing the two elastography methods, we encountered we found significant differences in term of liver stiffness only in children above the age of 12 years, noticing higher values of liver stiffness measured by TE (Fibroscan) versus 2D-SWE. Nevertheless, most-likely further studies on bigger samples of healthy pediatric patients would be useful in order to confirm our findings.

## Methods

### Ethics approval and informed consent

All the steps of the study were explained to both children and their parents/caregivers prior to their inclusion. Thus, the parents/caregivers signed the informed consent in behalf of their children, while all children gave their assent prior to the inclusion in our study. Our study was approved by the *Ethics Committee of the University of Medicine, Pharmacy, Sciences and Technology Târgu Mureș* (No 329/ November 17^th^ 2017), and it was performed according to the principles of the Helsinki Declaration.

### Study sample selection

We performed an observational study on 206 children aged between 3 to 18 years, in a Pediatric Tertiary Hospital, from November 2018 to July 2019. The inclusion criteria were: clinically healthy children, age between 3 and 18 years, normal weight (BMI Percentile > = 5 and <85), without an acute or chronic disorder, no recent history of any medication, normal laboratory parameters (complete cellular blood count, liver function parameters, C-reactive protein), children which were brought for a routine medical consult as a result of their parents/caregivers choice, and children with normal morphology and echogenicity of the liver parenchyma. The exclusion criteria consisted in age below 3 years due to their lack of compliance, modified laboratory parameters, abnormal hepatic ultrasound (US), acute or chronic diseases, children with obesity independently of the laboratory parameters or liver ultrasound aspect, but also children whose parents refused to participate in the study. All children were assessed on a one-day chart system. Thus, a thorough anamnesis and clinical exam along with anthropometric measurements were performed in all children. Those, who fulfilled the criteria regarding the anamnesis and clinical exam, underwent laboratory tests (complete cellular blood count, C-reactive protein, liver transaminases) and a normal US exam for the assessment of liver morphology and structure along with the other abdominal organs. All children that presented normal values of both laboratory parameters and US according to age and gender^39^ were finally included in our study.

Liver stiffness was assessed in all cases by 2 methods TE and 2D-SWE using Logiq S8 General Electric machine

(General Electric Healthcare, Wauwatosa, WI, USA) and a LOGIQ Shear Wave Elastography S8 XDclear 2.0 equivalent software (https://www.gehealthcare.com/products/ultrasound/logiq/logiq-s8-xdclear-2). TE was performed in all children with FibroScan module IPX0 (Manufacturer Echosens, Paris, France) using M probe. In order to achieve a value of liver stiffness as accurate as possible, 12 determinations were performed in each patient and the final value for each child was represented by their median (E Median), with an interquartile range (IQR) ≤ 30%.

2D-SWE was performed using a C1-6-D XDClear convex probe (Manufacturer General Electric Healthcare Company). The measurements were performed at approximately 1-2 cm under the hepatic capsule, on a region of interest of over 50% homogeneity, with a size of 0.5 cm. Similar to TE, 12 measurements were also obtained for 2D-SWE in each child, but we included in our study only the median of these 12 (E Median). Using 2D-SWE we also obtained 12 measurements for velocity concomitantly with LS, and we considered only the median of these values (V Median).

Both methods assessed the right liver lobe. Results were expressed in KPa for liver stiffness/elasticity in case of both TE and 2D-SWE, and m/s for velocity. All measurements were performed after a fasting period of approximately 6 hours, by a highly skilled physician with over 10 years in pediatric US and over 2 years in hepatic elastography,

### Statistical analysis

The statistical analysis comprised both descriptive statistics elements (mean, median, standard deviation, standard error, confidence interval 95%) and inferential statistics ones. The Shapiro-Wilk test was applied for determining the distribution of analyzed series of data. In order to compare the medians of liver stiffness obtained by 2D-SWE method, we applied t-Student test for unpaired data, t-Student test with Welch correction and ANOVA test, while for multiple comparisons, we applied Bonferroni test (post hoc).

In order to compare the medians of liver elasticity obtained through TE, we applied t-Student test for unpaired data, t-Student test with Welch correction, but also Mann-Whitney test, Kruskal-Wallis test, while for multiple comparisons we applied Dunn test (post hoc).

Spearman test was applied for correlation determination. The chosen significance threshold for p value was 0.05. The statistical analysis was performed using the GraphPad Prism trial variant program.

Intra-observer reliability was assessed based on Intraclass correlation coefficient (ICC) using SPSS statistical package version 15 (SPSS Inc, Chicago, IL). The analysis was based on a mean-rating, absolute-agreement, 2-way mixed-effects model. According to the guideline of Koo and Li^40^, ICC values less than 0.5 are indicative of poor reliability, values between 0.5 and 0.75 indicate moderate reliability, those between 0.75 and 0.9 indicate good reliability, while values greater than 0.90 indicate excellent reliability.
